# Notes on the Dematophytæ

**Published:** 1870-09

**Authors:** 


### Notes on the Dematophytæ.

The part played by fungi in causing disease is daily becoming more clearly recognised. Few now deny the vegetable nature of many cutaneous diseases, but as may be expected there are still very various and conflicting opinions in our modern school of dermatology. For example, no less than four dogmas are held to be indisputable by their various supporters, which may be divided, as correctly remarked by a reviewer in a late number of the *Glasgow Medical Journal,* into—1st. Those who agree with Erasmus Wilson, in denying altogether the parasitic nature of the so-called fungus, and, instead, hold the belief of a granular or phytiform degeneration. 2nd. Those who, like Hebra, consider that a parasite may occasionally be present, when it is an accidental complication. 3rd. Those who, with Tilbury Fox, believe that the cause of these diseases is the presence of a fungus, the differences observed in the appearances of each affection being due to the state of the growth of the cryptogan, soil and patient's constitution. 4th. Those who, with M'Call Anderson, think that soil and constitution have little influence upon the growth and development of the parasite. Every one has noticed the low forms of vegetable growths to be found on old bread, cheese, ink, books, &c. This fungus is known as the *Penicillium Glaucum*, and has certain analogies to another, the *Botrytes Bassiania*, which frequently attacks the silk-worm, producing the disease called *Muscardine*. The most favorable condition for the growth of these, as of all other fungi, is the presence of damp. Another circumstance favorable to their development, is the presence of a certain quantity of oxygen, which they readily absorb, giving off carbonic acid. It is a fact worthy of note that the vegetable moulds assume various forms, according to the localities and circumstances in which they are placed. For exaample Dr. Tilbury Fox placed a hair taken from a patch of tinea circinata in sugar and water, when it was observed that after a few days the spores become larger, and linked together after the manner of the achorion *mycelium* (favus); and he further informs us that "favus has been known to spring up in a patch of tinea circinata, and a clue to a proper explanation is afforded by the fact that the fungus takes on an active state of growth and sprouting from a favus cup. We must look for an explanation of the differences between the varieties of tinea, not so much in differences of fungus as of soil and seat upon which they grow." Any one engaged in a large cutaneous practice must have observed, especially on the body, the occasional occurrence of tinea circinata and favus. I have met with three such cases during the last two years, the notes of which have been published. I may, however, briefly mention that in one case, that of a boy, aged seven years, admitted February 8th, 1868; tinea circinata existed on the neck and chin. In the centre of one of the rings, which were all fading, there were several well-marked favus cups. On the chin the disease had assumed a tubercular character: the affection on this part, if covered with hair, would probabably have been called sycosis parasitica.

Many cases similar to the above have been recorded, and we must consider the occurrence together of the two diseases to be more than a mere coincidence. Mr. Law, professor of Veterinary Anatomy in Cornell University, when residing in Belfast, experimented upon some rabbits with the fungus taken from an apple, upon which the achorion had been transplanted. Mr. Law at the same time was experimenting on the inoculation of rabbits with tubercle, and, strange to say, it was only on two of those which had become tuberculous that the fungus flourished. Of course many failures took place before this desirable end was accomplished. The part where they had been inoculated, the inside of the ear, became, after some time, from one week to three, red and scaly, and took on the appearance of tinea circinata, Suffice it to say, that on microscopic examination, some of the scales and hair taken therefrom, first treated with liquor potassæ, and then with ether, showed numerous sporules. By this simple experiment, we can easily account for the occurrence of favus in mice and cats. The mice during their rambles come in contact with a fungus, most probably the *Penicillium Glaucum*, growing on old wood, for instance. In the natural state of affairs, they are caught by the cat, which then becomes attacked in turn, always on the fore paws and face, owing to the manner in which they kill their prey. I have seen a little girl with favus on the arm, owing to nursing a cat similarly affected. Dr. Purser, of Dublin, has published a case of tinea circinata occurring in a female, who contracted the disease from a cat, the subject of favus on one of her paws. No doubt the ordinary forms of "ringworm"—viz., tinea circinata, tonsurans and sycosis are due to the same parasite, the trichophyton tonsurans. This fact is conceded by all parties, and Dr. M'Call Anderson groups these diseases together in his last edition on Parasitic Diseases, under the name, tinea trichophytina. If we take a step further, and acknowledge the achorion to be a more natural form of the tricophyton, growing on a more favorable soil, I think that we will not be far from the truth. The researehes of Tulasne and de Barry. quoted by Dr. Tilbury Fox, have "contributed to the establishment of the doctrine of polymorphism, which implies that one fungus may pass through a cycle of development, and in its different stages give rise to many different forms, originally regarded as distinct species." —*Journal of Cutaneous Medicine, Sep.* 1870.

